# Trade-off between job losses and the spread of COVID-19 in Japan

**DOI:** 10.1007/s42973-021-00092-w

**Published:** 2021-08-25

**Authors:** Kisho Hoshi, Hiroyuki Kasahara, Ryo Makioka, Michio Suzuki, Satoshi Tanaka

**Affiliations:** 1 Vancouver School of Economics, UBC, Vancouver, Canada; 2 Research Institute of Economy, Trade and Industry (RIETI), Tokyo, Japan; 3 Economic and Social Research Institute (ESRI), Cabinet Office and Tohoku University, Sendai, Japan; 4 School of Economics, University of Queensland, Brisbane, Australia

**Keywords:** Mobility, SIRD model, Teleworkability, Panel data analysis

## Abstract

This paper quantitatively analyzes the trade-off between job losses and the spread of COVID-19 in Japan. We derive an empirical specification from the social planner’s resource constraint under the susceptible, infected, recovered, and deaths (SIRD) model and estimate how job losses and the case growth rate are related to people’s mobility using the Japanese prefecture-level panel data on confirmed cases, involuntary job losses, people’s mobility, and teleworkability. Our findings are summarized as follows. First, we find that a decrease in mobility driven by containment policies is associated with an increase in involuntary job separations, but the high teleworkability mitigates the negative effect of decreased mobility on job losses. Second, estimating how the case growth is related to people’s mobility and past cases, we find that the case growth rate is positively related to an increase in people’s mobility but negatively associated with past confirmed cases. Third, using these estimates, we provide a quantitative analysis of the trade-off between job losses and the number of confirmed cases. Taking Tokyo in July 2020 as a benchmark, we find that the cost of saving 1 job per month is 2.3 more confirmed cases per month in the short run of 1 month. When we consider a trade-off for 3 months from July to September of 2020, protecting 1 job per month requires 6.6 more confirmed cases per month. Therefore, the trade-off becomes worse substantially in the longer run of 3 months, reflecting the exponential case growth when the people’s mobility is high.

## Introduction

Containment policies, such as the declaration of a state of emergency, are the primary policy instruments to fight against the spread of COVID-19. While the declaration of a state of emergency and semi-emergency spread prevention measures help reducing coronavirus transmission, it may be accompanied by economic damage such as bankruptcies and increased unemployment, especially in the restaurant, retail and tourism industries. Managing the trade-off between the spread of infection and economic costs is one of the most hotly debated policy issues in this pandemic. However, existing empirical studies that quantitatively estimate this trade-off using the Japanese micro-level data are limited.

This paper empirically analyzes a trade-off relationship between job losses and the number of infections by considering a version of the susceptible, infected, recovered, and deaths (SIRD) model in which people’s movement determines both job losses and the spread of COVID-19. To highlight the essence of the trade-off between job losses and the spread of COVID-19, we consider the optimization problem of a social planner who maximizes the sum of discounted utilities given the technological and resource constraint on consumption and mortality by choosing the sequence of people’s mobilities. In the model, if people’s mobility increases, the growth rate of infections will increase, and the number of unemployed workers will decrease. We quantitatively analyze this trade-off relationship in Japan by examining how the growth rate of confirmed cases and the number of involuntary job losses change as people’s mobility goes up and down using the Japanese prefecture-level panel data.

**Fig. 1:**
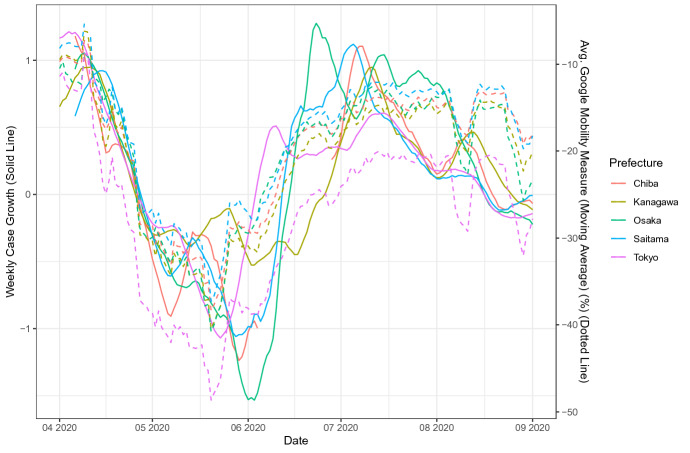
The weekly case growth and the mobility index for Chiba, Kanagawa, Osaka, Saitama, and Tokyo. The solid lines show the weekly case growth rates while the dotted line shows the mobility index defined by the weekly average of four Google mobility measures lagged by 14 days. Different colours represent different prefectures, where the red is for Chiba, the dark green is for Kanagawa, the light green is for Osaka, the blue is for Saitama, and the purple is for Tokyo

**Fig. 2:**
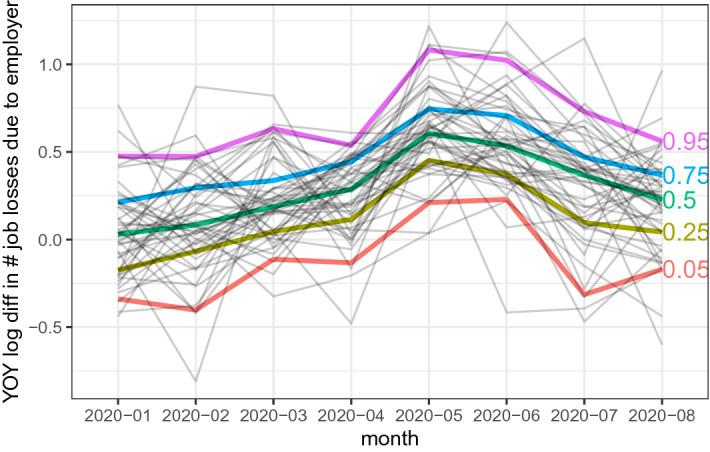
The involuntary job separations, Jan 2020–August 2020. The above figure shows year-over-year log difference in the number of job losses due to employer reasons for 47 prefectures in Japan. We use the number of “involuntary job separations due to employer” from the Ministry of Health, Labour and Welfare’s Monthly Report on the Employment Insurance Programs (*Koyou-Hoken-Jigyou-Geppou*)

The time-series association of people’s mobility with the case growth rate and job losses is apparent in the data. Figure 1 shows the evolution of weekly confirmed case growths and the weekly mobility index from Google Mobility Reports for Chiba, Kanagawa, Osaka, Saitama, and Tokyo, where the solid and dotted lines show the confirmed case growth and the 2-weeks lagged mobility index, respectively. Both the case growth and the lagged mobility index simultaneously dropped in May and June, leading to a positive time-series correlation. Figure 2 shows the movement of year-over-year log differences of job losses for 47 prefectures during the time of the COVID-19 spread in Japan, where the solid colored lines represent the 5, 25, 50, 75, and 95 percentile values. The numbers of involuntary job losses have started rising gradually from March 2020, and then peaked around May or June 2020, which coincides with the timing of a significant drop in people’s mobility. Sections 4.1 and 5.1 discuss the construction of the variables in the details.

In our analysis, we derive an empirical specification from the social planner’s resource constraint and estimate how job losses are related to people’s mobility using the monthly panel data across prefectures, where we incorporate the difference in the occupation/industry structures across prefectures using the measures of teleworkability. We also derive an empirical specification from the SIRD model on how the case growth is affected by people’s mobility and past confirmed cases and estimate the impact of people’s mobility on the case growth using the daily panel data on case growth and people’s mobility. Combining these two estimates, we quantify the trade-off between job losses and the number of cases as the social planner changes the mobility level. The constructed trade-off from the estimated regressions can be interpreted as an empirical analog of the resource constraint in the social planner’s problem.

The main empirical findings are summarized as follows. First, our panel data regression analysis indicates that a decrease in mobility is associated with an increase in involuntary job separations, but the teleworkability can mitigate the negative effect of decreased mobility on job losses in a high-teleworkable prefecture (e.g., Tokyo). This result suggests that teleworkability played an important role in protecting jobs in Tokyo. Using the containment policy index as an instrument for the mobility index, the instrumental variable regression finds similar results. Furthermore, when we use the containment policy index in place of the mobility measure, we find that containment policies increase job losses, but the teleworkability mitigates such a negative impact.

Second, estimating how the case growth is related to people’s mobility and past cases, we find that increasing people’s mobility increases the growth rate of confirmed cases with two weeks lag. We also find that the case growth rates are negatively associated with the past cases. These findings are consistent with those from the existing studies (e.g., Chernozhukov et al. 2021b). An increase in mobility increases the contact rate among people, leading to a higher transmission rate of SARS-Cov-2. The negative estimate of past cases suggests that people take more preventive actions (e.g., voluntary social distancing, mask-wearing, and handwashing) to respond to the new information on increasing transmission risks, which decreases the infection probability conditional on meeting with infectious people.

Third, using the estimated result on the job loss regression and the case growth regression, we provide a quantitative analysis of the trade-off between job losses and the number of confirmed cases, holding other conditions constant. Taking Tokyo in July 2020 as a benchmark, we find that the cost of saving 1 job loss per month is 2.3 more confirmed cases within a month in the short run of one month. However, when we consider a longer-run trade-off for three months from July to September 2020, the trade-off becomes worse substantially: on average over three months, protecting 1 job per month requires 6.6 more confirmed cases per month in the longer run of three months. Our analysis indicates that keeping low job losses for a more extended period requires a more significant number of cumulative cases because of the exponential case growth. Furthermore, in a counterfactual situation where the number of weekly cases at the beginning of July 2020 were 2520—the highest number of weekly cases recorded in January of 2021 in Tokyo—instead of the actual number of weekly cases, 67, protecting 1 job requires 134 confirmed cases. Therefore, protecting jobs is quite costly when the number of cases is very high. We also find that the trade-off in Tokyo is much better than in other prefectures such as Aichi because the job losses caused by a drop in mobility are much less in Tokyo than in other prefectures given that many jobs in Tokyo are teleworkable from home.

This paper is related to the recent literature that presents theoretical frameworks for the economic and health trade-offs. Atkeson (2020) is one of the early papers that extends the SIR model of Kermack and McKendrick (1927), and analyzes economic losses after the new COVID-19 outbreak. Eichenbaum et al. (2020) combine the SIRD model with a standard macroeconomic model to study the trade-offs between economic losses and COVID-19 cases. They derive an optimal non-pharmaceutical policy, which sacrifices economic activities to maximize social welfare, reducing the damage from infection. Other studies that have used the SIRD-macro model to study the economic and health trade-offs include Krueger et al. (2020), Jones et al. (2020), and Kaplan et al. (2020).

While several papers have developed theoretical macroeconomic frameworks on the economic-health trade-off, few existing papers have analyzed the trade-off empirically using the micro-level data. Indeed, the empirical literature on the factors contributing to COVID-19 case growth and COVID-19’s economic impact has developed separately and is disconnected from each other. In the former literature, several papers have examined the association between non-pharmaceutical interventions, social distancing behavior and COVID-19 cases (Hsiang et al. 2020; Courtemanche et al. 2020; Abouk and Heydari 2021; Gupta et al. 2020; Maloney and Taskin 2020; Andersen 2020; Pei et al. 2020; Chernozhukov et al. 2021b). Among those studies, Chernozhukov et al. (2021b) is closest to our paper, as they estimate the impact of various policies adopted by US states on the growth rates of confirmed Covid-19 cases and deaths, as well as social distancing behavior measured by Google Mobility Reports.

The latter literature analyzes the effect of COVID-19 on labor markets. Using the Current Population Survey in the US, several papers show that the negative impact of COVID-19 on employment was large for people who could not work from home, those in high-physical-proximity jobs, Hispanic, younger workers, those with high school degrees and some college, females, and workers in “non-essential” industries (Gupta et al. 2020a; Mongey et al. 2020; Montenovo et al. 2020; Albanesi and Kim 2021; Lee et al. 2021). Similar patterns are observed in different countries, such as Guven et al. (2020) for Australia and Casarico and Lattanzio (2020)) for Italy.^1^ This paper contributes to the literature by providing a quantitative analysis of the trade-off between job losses and the number of confirmed cases by connecting the results of the job loss regression and the case growth regression.

Finally, our paper is also related to the studies on the COVID-19 effect in Japan. For example, Kikuchi et al. (2021), Kawata (2020), Fukai et al. (2021), and Hoshi et al. (2021) analyze the effect of COVID-19 on labor markets. Our paper differs from these papers by focusing on a trade-off relationship between job losses and the number of infections.

The remainder of this paper is as follows. Section 2 presents a social planner’s dynamic decision problem. Section 3 derives empirical specifications from the social planner’s resource constraint. Sections 4 and 5 estimate the effect of policy-driven mobility changes on job losses and the growth of newly confirmed cases. Section 6 analyzes the empirical trade-off between job losses and confirmed cases in Japan. Section 7 concludes.

## Trade-off between job losses and the spread of COVID-19 from the viewpoint of the SIRD model

### Social planner’s problem

To model the trade-off, we pay particular attention to the role of people’s mobility. On the one hand, an increase in people’s mobility may induce the spread of COVID-19 by inducing a higher transmission of SARS-CoV-2. But, on the other hand, people’s mobility is also associated with the aggregate output through supply-side and demand-side factors during the pandemic period. In particular, a reduction in people’s mobility induced by containment policies and people’s voluntary behavioral change reduces the aggregate output. The negative relationship between mobility and output arises because consuming some goods requires people’s movement (e.g., shopping, live entertainment, eating at restaurants, and staying at hotels); furthermore, some types of work are impossible to perform without being at the workplace (e.g., serving at restaurants).

To capture the supply-side relationship between the people’s mobility and the aggregate output production in reduced form, we assume that the output of the economy is determined by the Leontief production function with labor input and “mobility” input:

1 $$\begin{aligned} { Y_t=\min \{L_t,\varphi (M_t)\}}, \end{aligned}$$

where $Y_t$ is the aggregate output, $L_t$ is employment, $M_t$ is the amount of people’s mobility, $\varphi (M)$ is a strictly increasing function of *M*.^2^

It is also reasonable to assume that, in the pandemic, a reduction of mobility affects consumption because consuming some goods (e.g., eating at restaurants) requires mobility. To capture this demand-side relationship, we assume that the aggregate consumption is determined by the available aggregate output *Y* and the mobility $M_t$ as:

2 $$\begin{aligned} { C_t=\min \{Y_t,\psi (M_t)\}}, \end{aligned}$$

where $C_t$ is the aggregate consumption and $\psi (M)$ is a strictly increasing function of *M*.

The economy is initially endowed with $\bar{N}$ workers, where each worker supplies one unit of labor. Let $N_t:=\bar{N}-D_t$ denote the number of workers survived at time *t*, where $D_t$ is the cumulative deaths at *t*. The labor supply is restricted by the surviving population so that $L_t\le N_t$. The social planner’s utility flow in each period depends on the number of surviving workers and the consumption of goods and is given by $U(N_t,C_t)$. Here, we assume that $\partial U(N_t,C_t)/\partial N_t>0$ to reflect the disutility from deaths.

The spread of COVID-19 leads to death among workers, which leads to a decrease in social planner’s utility flow from a reduction in $N_t$. The dynamics of deaths and infections is determined by the susceptible–infectious–recovered–deceased (SIRD) model as:

3 $$\begin{aligned} S_{t+1}&= S_t -\frac{ S_t}{\bar{N}} \beta _t({ M_t},I_{t-1}) I_t, \end{aligned}$$

4 $$\begin{aligned} I_{t+1}&= (1-\gamma ) I_t +\frac{ S_t}{\bar{N}} \beta _t({ M_t},I_{t-1}) I_t, \end{aligned}$$

5 $$\begin{aligned} R_{t+1}&= R_t + \pi _r { I _{t}}, \end{aligned}$$

6 $$\begin{aligned} { D_{t+1} }&= D_t + \pi _d { I_{t}}, \end{aligned}$$

where $S_t$, $I_t$, $R_t$, and $D_t$ denote the number of susceptible, infected, recovered, and deceased individuals at *t*. $\pi _r$ and $\pi _d$ are the probability of recovery and death conditioning on being infected, respectively, and $\gamma :=\pi _r+\pi _d$.

The variable $\beta _t(M_t,I_{t-1})$ denotes the infection rate at *t* that depends on both the mobility $M_t$ and the lagged infection. We assume that increasing mobility leads to a higher infection rate so that $\partial \beta _t(M_t,I_{t-1})/\partial M_t>0$ while an increase in the lagged infection leads to a lower infection rate, i.e., $\partial \beta _t(M_t,I_{t-1})/\partial I_{t-1}<0$. The positive effect of mobility on infection rates reflects that increasing mobility increases contact rates among people, leading to a higher virus transmission rate. On the other hand, the negative effect of the past infections on the current infection rate is interpreted as follows. As people are informed about the high number of past infections, being aware of the higher transmission risk, people voluntarily take preventive actions (social distancing, mask-wearing, and frequent hand washing), which reduces the probability of being infected conditional on contacting infectious people.

The social planner solves the dynamic optimization problem of maximizing the sum of discounted utility flows given the state $\mathbf{X}_{t} := (S_t, I_t, D_t,I_{t-1})$ with $R_t= \bar{N} -S_t - I_t - D_t$ by choosing a sequence of mobilities $\{M_{t+s}\}_{s=0}^{\infty }$ as

7 $$\begin{aligned} V(\mathbf{X}_{t})&= \max _{\{{ M_{t+s},L_{t+s}}\}_{s=0}^{\infty }} \sum _{s=0}^\infty \delta ^s U(N_{t+s},C_{t+s}) \nonumber \\&\text {s.t.}C_{t+s} = \min \{Y_{t+s},\psi (M_{t+s})\},\quad Y_{t+s} = \min \{L_{t+s},\varphi (M_{t+s})\}\nonumber \\&L_{t+s} \le N_{t+s} = \bar{N} - D_{t+s}=S_{t+s}+I_{t+s}+R_{t+s},\nonumber \\&\text {Eq.}\ \text {(3)-(6)}\text { hold for every } t. \end{aligned}$$

When there is no pandemic, mobility does not need to be restricted, and all workers will engage in production activities, and people freely visit various places for consumption activities so that the output is determined as $C_t=Y_t=L_t=N_t$ for all *t*. However, in the pandemic period, the social planner may choose to restrict the mobility $M_t$ to reduce the spread of COVID-19. Because an increase in mobility incurs the cost of future deaths, given (1)–(2), the social planner will choose the mobility $M_t$ and the labor input $L_t$ so that

8 $$\begin{aligned} C_t=Y_t=L_t=\min \{\varphi (M_t),\psi (M_t)\}:=\bar{\varphi }(M_t), \end{aligned}$$

where $\bar{\varphi }(M_t):=\min \{\varphi (M_t),\psi (M_t)\}$ is strictly increasing in $M_t$.^3^ (8) holds if the number of infectious workers is strictly positive so that increasing mobility leads to future disutility from an increase in future deaths. Furthermore, when the mobility is sufficiently restricted, the mobility determines the output, and some workers become unemployed: $L_t<N_t$.

The derivative of $\varphi (M_t)$ captures how sensitive the aggregate output is for a change in the mobilities. Similarly, the derivative of $\psi (M_t)$ captures how sensitive the aggregate demand is for a change in mobility because consumption of some goods requires people’s mobility. From the labor supply-side viewpoint, the derivative, $\partial \varphi (M_t)/\partial M_t$, captures the degree of teleworkability. For example, suppose the economy heavily relies on the sectors that cannot do remote work (e.g., restaurants). In that case, a forced reduction in mobility may lead to a large decrease in output because output cannot be produced without people moving to the workplace. In contrast, if the economy consists of the sectors in which work can be done from home (e.g., online game industry), decreasing mobility may not reduce output. The supply-side measure of teleworkability is likely to be positively correlated with the demand-side “teleconsumability”—the extent to which goods can be consumed from home. When restaurants and hotels are the primary industries in the economy, both teleworkability and teleconsumability would be low.^4^

To simplify our analysis, given (8), we also assume that $U(N_t,Y_t)$ is homogenous of degree one, strictly increasing, and concave so that $U(N_t,Y_t)=N_t U(1,y_t)=N_t u(y_t)$, where $y_t:=Y_t/N_t$ is the per-capita consumption and $u(y_t):=U(1,y_t)$ is the utility flow for each worker with $u'(y)>0$ and $u''(y)<0$. Therefore, the social planner’s utility flow is given by the number of surviving workers times the utility flow for each surviving worker represented by $u(y_t)$. This utility function implicitly assumes that each worker receives no utility from leisure.

Under these assumptions, the social planner’s dynamic optimization problem is written as

9 $$\begin{aligned} V(\mathbf{X}_{t}) = \max _{\{{ M_{t+s}}\}_{s=0}^{\infty }}&\sum _{s=0}^\infty \delta ^s (\bar{N} -D_{t+s}) u(y_{t+s}) \nonumber \\ \text {s.t.}&\quad y_{t+s} = \frac{L_{t+s}}{\bar{N}-D_{t+s}},\quad L_{t+s}= \bar{\varphi }(M_{t+s}),\nonumber \\&\quad \text {Eq.}\ \text {(3)-(6)}\ \text {hold for every } t, \end{aligned}$$

and the corresponding Bellman equation is given by

10 $$\begin{aligned} V(\mathbf{X}_{t}) = \max _{M_{t}}&\ (\bar{N} -D_{t}) u(y_{t}) + \delta V( \mathbf{X}_{t+1}) \nonumber \\ \text {s.t.}&\quad y_{t} = \frac{\bar{\varphi }(M_{t})}{\bar{N}-D_{t}}, \quad \text {Eq.}\ \text {(3)-(6)} \text { hold.} \end{aligned}$$

The first-order condition for the Bellman equation (10) gives some insight on the trade-off between the utility flow associated with the current consumption and the future loss associated with future infections and deaths:

11 $$\begin{aligned} { u'(y_t) \bar{\varphi }'(M_t)}=&\quad - \delta \left( {\frac{\partial V(\mathbf{X}_{t+1})}{\partial I_{t+1}} } { - \frac{\partial V(\mathbf{X}_{t+1})}{\partial S_{t+1}}} + { \pi _d \frac{\partial V(\mathbf{X}_{t+1})}{\partial D_{t+1}}} \right) \frac{\partial I_{t+1}}{\partial M_t} \end{aligned}$$

with

$$\begin{aligned} \frac{\partial I_{t+1}}{\partial M_t}=\frac{ S_t}{\bar{N}}\frac{\partial \beta _t(M_t,I_{t-1})}{\partial M_t} I_t>0, \end{aligned}$$

where each term in (11) can be interpreted as follows:

$$\begin{aligned} { u'(y_t) \varphi '(M_t)}>0:&\text {gain in utility flow from a higher current output}\\ {\frac{\partial V(\mathbf{X}_{t+1})}{\partial I_{t+1}}<0} :&\text {infectious } \uparrow \Rightarrow \text { future infections/deaths } \uparrow \\ { - \frac{\partial V(\mathbf{X}_{t+1})}{\partial S_{t+1}} >0}:&\text {susceptible } \downarrow \Rightarrow \text { future infections/deaths } \downarrow \\ { \pi _d \frac{\partial V(\mathbf{X}_{t+1})}{\partial D_{t+1}} <0}:&\text {disutility from deaths}. \end{aligned}$$

An increase in mobility increases the output, which increases the current utility flow. On the other hand, a higher level of mobility leads to a higher level of future infections, leading to a larger number of deaths in the future.

### Trade-off between job losses and infections

From (4) and (8), we may relate the mobility $M_t$ to the number of unemployed workers $N_t -L_t$ and the growth rate of infectious workers $(I_{t+1}-I_t)/I_t$ as:

12 $$\begin{aligned} \frac{I_{t+1}-I_{t}}{I_{t}}&= - \gamma + \frac{ S_t}{\bar{N}} \beta _t({M_t},I_{t-1}), \end{aligned}$$

13 $$\begin{aligned} N_t-L_t&= N_t-\bar{\varphi }({ M_t}), \end{aligned}$$

where $\frac{ S_t}{\bar{N}} \approx 1$ in the early pandemic period. Equations (12) and (13) motivate our empirical specifications. In (12), an increase in the mobility increases the growth rate of infections, $\frac{I_{t+1}-I_{t}}{I_{t}}$. On the other hand, (13) indicates that a decline in mobility leads to job losses denoted by $N_t-L_t$.

By eliminating the mobility $M_t$ from (12) and (13), we obtain

14 $$\begin{aligned} I_{t+1} = \left\{ 1-\gamma + \frac{ S_t}{\bar{N}}\beta _t(\bar{\varphi }^{-1}(L_t),I_{t-1})\right\} I_t. \end{aligned}$$

Given the value of $S_t$, $I_t$, and $I_{t-1}$, this equation represents a pair of values $(I_{t+1},L_t)$ which a social planner can choose from in the short-run (i.e., within one period).

By repeated substitutions, we may also characterize the longer-run trade-off over multiple periods between a sequence of the number of infections $\{I_{t+s}\}_{s=1}^J$ and a sequence of employment $\{L_{t+s}\}_{s=0}^{J-1}$ as

15 $$\begin{aligned} I_{t+J} = \prod _{s=0}^{J-1} \left\{ 1-\gamma + \frac{ S_{t+s}}{\bar{N}}\beta _t(\bar{\varphi }^{-1}(L_{t+s}),I_{t+s-1})\right\} I_t\quad \text {for } J=1,2,\ldots . \end{aligned}$$

Equation (15) has an important implication on the nature of trade-off in an extended period between the number of infections and employed workers. In particular, when the term inside the bracket on the right-hand side of (15) is larger than one, the number of infections grows exponentially as the time horizon *J* increases. Therefore, keeping the high number of employed workers for a long period during the pandemic may be very costly in terms of the number of infections and deaths.

Another insight we obtain from (14)–(15) is that the trade-off relationship between the number of jobs and the number of cases critically depends on the initial level of cases. If the number of current cases $I_t$ is ten times as large, then protecting 1 job may require almost ten times as large of confirmed cases in the next period. This is because the SIRD model implies that increasing mobility affects the growth rate of infections rather than the level of infections and, therefore, the trade-off relationship between the number of jobs and the number of cases is heavily influenced by the initial level of infections.

## Empirical specifications

We derive our empirical specifications from (12) and (13). The job loss regression is obtained from (13) by specifying $\varphi ({ M_t})$ as a linear function of the mobility variable, its interaction with the teleworkability index, and other controls as

16 $$\begin{aligned} \text {Job Loss}_{ij}= & {} \beta _1 \text {Mobility}_{ij} + \beta _2 \text {Mobility}_{ij}\times \text {Tel}_{i} + \gamma \text {Tel}_{i}+ \alpha 'X_{i}+\epsilon _{ij} \end{aligned}$$

with

$$\begin{aligned} \text {Job Loss}_{ij}:= \ln \left( \frac{Y_{i,j,2020}}{Y_{i,j,2019}}\right) , \end{aligned}$$

where *i* is prefecture and *j* is month. The outcome variable $\text {Job Loss}_{ij}$ is the log of the monthly number of job losses due to employer in month *j* of year 2020 relative to the corresponding value of year 2019, where $Y_{i,j, year}$ is the number of job losses due to employer in month *j* of the corresponding year. $\text {Mobility}_{ij}$ is the monthly average value of Google mobility variable while $\text {Tel}_i$ is the teleworkability index of prefecture *i*. $X_i$ includes monthly dummies and the prefecture-specific covariates including the log of GDP per capita, poverty rate, elderly rate, and population density. In the above equation, we interact the mobility with the telework index for the prefecture. This interaction term is supposed to capture the extent to which teleworkability mitigates the effects of COVID-19 on job losses.

In estimating the job loss regression (16), the endogeneity of the mobility index is a major concern. While the regression includes month dummies and various prefecture-specific controls, unobserved prefecture-month specific supply or demand shocks correlated with mobility index may bias the estimates. The mobility should depend on various confounders, such as regional demographics, types of occupations, industry structure, people’s perception and risk attitude toward infections, which observed controls may not fully control.

As an attempt to mitigate the endogeneity concern, we consider an alternative specification in which we use the policy index in (20) in place of the mobility index. Furthermore, we also implement the Instrumental Variable (IV) regression to estimate (16) using the policy index as an instrument for the mobility index under the identifying assumption that the policy index is correlated with the mobility index but uncorrelated with the error term $\epsilon _{ij}$ in (16). A potential concern here is the violation of exclusion restriction, i.e., the policy index may also be potentially correlated with the error term $\epsilon _{ij}$ if the policy choice is affected by local economic conditions such as job losses. For this reason, the result of our study should be interpreted with great caution.

Following the research design in Chernozhukov et al. (2021b), in (12), we approximate the growth rate of infections by the log difference in weekly confirmed cases and specifying $\frac{ S_t}{\bar{N}} \beta _t({M_t},I_{t-1})$ as a linear function of mobility variable, past cases, past case growth, and other covariates as:

17 $$\begin{aligned} \varDelta \log \text {Case}_{it} = \alpha \text {Mobility}_{i,t-14} + \mu _1 \log \text {Case}_{i,t-14} + \mu _2 \varDelta \log \text {Case}_{i,t-14} + \delta _Y 'X_{it} + \varepsilon ^y_{it}, \end{aligned}$$

where *i* is prefecture and *t* is day. The outcome variable $\varDelta \log \text {Case}_{it}=\log \text {Case}_{it} - \log \text {Case}_{i,t-7}$ is the log-difference over 7 days in weekly confirmed cases with $\text {Case}_{it}$ denoting the number of confirmed cases from day $t-6$ to *t*. We set the value of $\log \text {Case}_{it}$ to $-1$ for the observation with zero weekly cases. $\text {Mobility}_{i,t-14}$, $\log \text {Case}_{i,t-14}$, and $\varDelta \log \text {Case}_{i,t-14} =\log \text {Case}_{i,t-14}-\log \text {Case}_{i,t-21}$ are the past Google mobility variable, the log of past weekly cases, and the log difference of past weekly cases, respectively, where they are lagged by 14 days to capture the time lag between infection and case reporting. $X_{it}$ includes the number of tests, prefecture-level characteristics, monthly dummies, and the Golden Week dummies lagged by 14 days.^5^

We also examine how the mobility is affected by the containment policies implemented by the prefecture governments by estimating the following regression:

18 $$\begin{aligned} \text {Mobility}_{it} = \beta \text {Policy}_{it} + \gamma _1 \log \text {Case}_{i,t-14} + \gamma _2 \varDelta \log \text {Case}_{i,t-14} + \delta _M 'X_{it} + \varepsilon ^m_{it}, \end{aligned}$$

where the outcome variable is the mobility index $M_{it}$. The main explanatory variable of interest is the index for containment policies, $\text {Policy}_{it}$. The log of weekly cases and their log differences lagged by 14 days capture the effect of perceived transmission risk on people’s mobility while $X_{it}$ includes prefecture-level characteristics, monthly dummies, and the Golden Week dummies.

In our empirical analysis, we estimate (16) and (17) and then using the estimates, we compute the empirical analogues of (14) and (15) to illustrate the trade-off between the number of confirmed cases and job losses in the short-run within one month as well as in the longer run over 3 months.

## The job loss regression

To quantify the trade-off between job losses and the spread of COVID-19, we first estimate the relationship between people’s movement and job losses using the prefecture-month panel data.

### Prefecture-month panel data

We construct the prefecture-month panel data for Japan from February to August 2020 to estimate the regression specification (16).

For the number of job losses, we use the number of “involuntary job separations due to employer” from the Ministry of Health, Labour and Welfare’s Monthly Report on the Employment Insurance Programs (*Koyou-Hoken-Jigyou-Geppou*). This is the official statistic of the number of the unemployment flow of workers after an involuntary separation. One limitation of this data is that it covers workers with employment insurance only, thus likely missing job losses of non-regular, part-time workers. Figure 2 in the introduction section shows that the numbers of involuntary job losses have started rising gradually from March 2020 and then peaked at either May or June 2020. The figure also suggests a large variation in the movements of involuntary job losses over time across prefectures.

Our mobility index is created from Google’s COVID-19 Community Mobility Reports.^6^ Google provides people’s visits to places over time by geography, across different categories of places such as retail and recreation, groceries and pharmacies, parks, transit stations, workplaces, and residential. The reports are available daily for each prefecture in Japan. Our mobility index for prefecture *i* in month *j* is created by taking the average of the corresponding workplace, retail, grocery, and transit mobility measures from Google’s Community Mobility Reports, where each of four mobility measures is its monthly average measure within month *j* in prefecture *i*:

19 $$\begin{aligned} \text {Mobility}_{ij} = \frac{\text {Workplaces}_{ij}+\text {Retail}_{ij}+\text {Grocery}_{ij}+\text {Transit}_{ij}}{4}. \end{aligned}$$

**Fig. 3:**
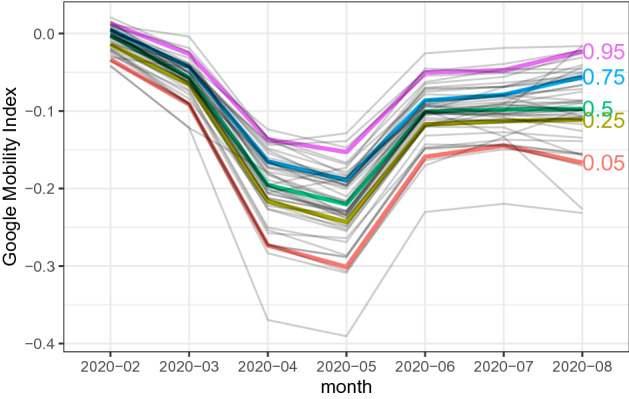
The mobility index, Feb 2020–August 2020. The above figure shows our mobility index for 47 prefectures in Japan. We use Google’s COVID-19 Community Mobility Reports. Our mobility index is created by taking the average of the workplace, retail, gorcery, and traisit mobility measures. Each of these measures are shown in Fig. 11 in Appendix

We chose these four measures as they are supposed to capture major economic activities, which are greatly affected by the spread of COVID-19. Figure 3 shows the movement of the mobility index for 47 prefectures in Japan during the time of the COVID-19 spread. As shown in the figure, the mobility index has dropped greatly in April and May 2020 when the first wave of COVID-19 hit Japan, while the extent of the drop differs across prefectures.

**Fig. 4:**
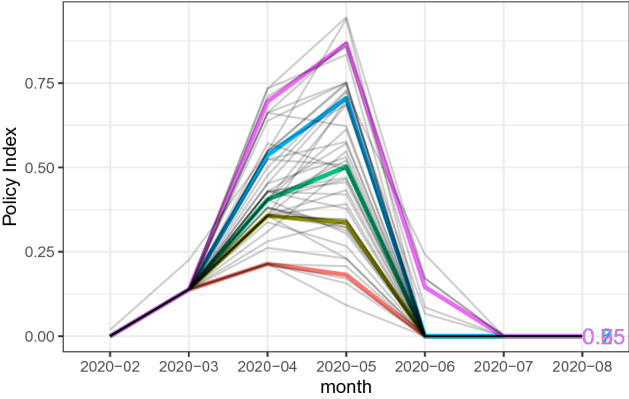
The policy index, Feb 2020–August 2020. The above figure shows our policy index for 47 prefectures in Japan. We create our policy index by taking the average of the seven policy dummy variables. Those dummy variables include status of emergency declaration, closure of museums, closure of schools, closure of commercial stores, closure of restaurants and bars, and closure of nightclubs

We create our policy index by taking the average of the seven policy dummy variables as

20 $$\begin{aligned} \text {Policy}_{ij} = \frac{\text {Emergency}_{ij}+\text {School}_{ij}+\text {Bars}_{ij}+\text {Commercial}_{ij}+\text {Movie}_{ij}+\text {Museum}_{ij}+\text {Nightclub}_{ij}}{7}, \end{aligned}$$

where the seven policy dummy variables include state of emergency declaration, closures of schools, bars/restaurants, commercial stores, movie theaters, museums, and nightclubs. We collect the information from newspapers and other public media, and record the dates of policy changes. These records are available on our website.^7^ Figure 4 shows the movement of the policy index for 47 prefectures in Japan during the time of the COVID-19 spread. As shown in the figure, most prefectures had implemented their non-pharmaceutical intervention policies in March, April, and May in 2020. On the other hand, the timing and the extent of these policy responses differ across prefectures.

### Teleworkability

**Fig. 5:**
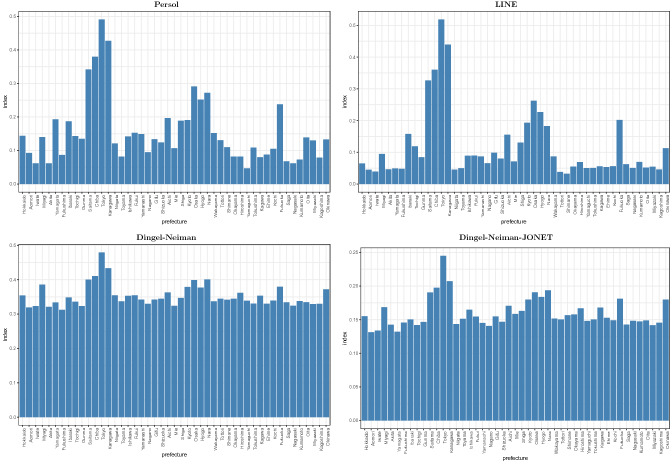
The teleworkability indices across 47 prefectures. The each panel of the above figure shows our teleworkability indexes for 47 prefectures in Japan. ‘Persol’ and ‘LINE’ indexes are based on the actual telework hours from the two different surveys, while ‘Dingel-Neiman’ and ‘Dingel-Neiman-JONET’ are created based on the task contents of each occupation

For the teleworkability index, we consider four different measures. The first two measures are based on actual telework hours data during the COVID-19 pandemic. The first measure is from the survey for regular workers conducted on April 12 and 13 in 2020 by Persol Research and Consulting Co., Ltd (Persol), a private company located in Tokyo. The second measure is from a similar survey conducted by LINE Co. on April 10 through 12 in 2020. In contrast to Persol’s survey, this survey interviews office workers only. Both Persol and LINE aggregate their survey data to the prefecture level, which we use as our measure of teleworkability index.

The third and the fourth measures are created based on the occupational task approach as in Dingel and Neiman (2020). Dingel and Neiman (2020) classify the feasibility of working at home for all occupations using the O*NET database, created by the US Department of Labor. They use two sets of surveys from O*NET. One is called the Work Context Questionnaire, which asks questions to capture the “physical and social factors that influence the nature of work,” such as interpersonal relationships, physical work conditions, and structural job characteristics. The second is called the Generalized Work Activities Questionnaire, which includes questions aiming to capture the “general types of job behaviors occurring on multiple jobs,” such as the input of information, interaction with others, mental processes, and work output. With these surveys, they determine whether occupational tasks can be performed at home, by considering, for example, frequency in the usage of emails, a requirement for physical activities, etc.

Our third teleworkability measure is constructed by mapping Digel and Neiman’s measure to the occupational classification in the Japanese Population Census data and aggregated them to prefecture level using the number of workers in each occupation as weights.^8^ Furthermore, we create the fourth measure of teleworkability based on the Japanese ONET (JONET) data, which is the equivalent of the US O*NET dataset, created by the Ministry of Health, Labour and Welfare in Japan.

As shown in Fig. 5, these four indexes look highly correlated with the highest value for Tokyo, even though they are based on different approaches and different data sources. In our job loss regression, we estimate the effects of the interaction between the mobility and teleworkability indexes to examine the extent to which the teleworkability mitigates the negative impact of mobility drops on involuntary job losses.

### Regression results

**Table 1:** Job loss regressions using a mobility index

	Dependent variable: involuntary job separations			
	(1)	(2)	(3)	(4)
Mobility$$_{i,j}$$	− 0.775$$^{*}$$ (0.457)	− 2.753$$^{**}$$ (1.082)		
Policy$$_{i,j}$$			0.174 (0.120)	0.579$$^{**}$$ (0.292)
Persol$$_{i}$$	− 0.556$$^{**}$$ (0.277)	−0.232 (0.256)	− 0.504$$^{**}$$ (0.257)	− 0.375 (0.247)
Mobility$$_{i,j}\times$$ Persol$$_{i}$$		3.175$$^{***}$$ (1.211)		
Policy$_{i,j}\times$ Persol$_{i}$				− 0.842$^{*}$ (0.469)
Observations	282	282	282	282
$R^{2}$	0.485	0.497	0.485	0.494

All regressions include prefecture-specific controls and month dummies and are weighted by prefecture population. Standard errors clustered at the prefecture level are shown in parentheses

$$^{*}p<0.1$$; $$^{**}p<0.05$$; $$^{***}p < 0.01$$

**Table 2:** Job loss regressions with alternative teleworkability measures

	Dependent variable: involuntary job separations					
	Line	DN	DN-JONET	Line	DN	DN-JONET
	(1)	(2)	(3)	(4)	(5)	(6)
Mobility$$_{i,j}$$	− 2.773$$^{**}$$ (1.101)	− 7.275$$^{***}$$ (2.492)	− 4.015$$^{*}$$ (2.082)			
Policy$$_{i,j}$$				0.507$$^{*}$$ (0.278)	1.606$$^{**}$$ (0.675)	0.898$$^{*}$$ (0.535)
Tel$_{i}$	− 0.282 (0.233)	− 0.740 (1.391)	− 0.022 (1.047)	−0.381$^{*}$ (0.225)	− 1.679 (1.234)	− 0.284 (0.972)
Mobility$$_{i,j}\times$$ Tel$$_{i}$$	2.786$$^{**}$$ (1.114)	21.485$$^{***}$$ (7.378)	8.544$$^{**}$$ (4.248)			
Policy$$_{i,j}\times$$ Tel$$_{i}$$				− 0.676 (0.417)	− 5.271$$^{**}$$ (2.273)	− 2.190$$^{*}$$ (1.278)
Observations	282	282	282	282	282	282
$R^{2}$	0.501	0.499	0.479	0.496	0.493	0.478

All regressions include prefecture-specific controls and month dummies and are weighted by prefecture population. Standard errors clustered at the prefecture level are shown in parentheses

$$^{*}p<0.1$$; $$^{**}p<0.05$$; $$^{***}p<0.01$$

**Table 3:** Job loss regressions with the policy index as an instrument

	Dependent variable: involuntary job separations			
	Persol	Line	DN	DN-JONET
	(1)	(2)	(3)	(4)
Mobility$$_{i,j}$$	− 7.384$$^{**}$$ (3.725)	− 6.568$$^{*}$$ (3.616)	− 10.884$$^{**}$$ (4.340)	−8.706 (5.627)
Mobility$$_{i,j}\times Tel_{i}$$	7.172$$^{**}$$ (3.432)	5.719$$^{*}$$ (2.994)	31.141$$^{***}$$ (11.622)	16.316$$^{*}$$ (9.668)
Tel$_{i}$	− 0.165 (0.258)	− 0.261 (0.216)	− 0.210 (1.246)	− 0.184 (1.003)
Observations	282	282	282	282
$R^{2}$	0.452	0.469	0.494	0.463
F stat: Mobility	32.098	32.582	53.065	118.034
F stat: Mobility $\times$ Tel	204.611	181.496	69.617	369.744

All regressions include prefecture-specific controls and month dummies and are weighted by prefecture population. The rows “F-stat: Mobility” and “F-stat: Mobility $\times$ Tel” report the first-stage F-statistics on the relevance of instruments for Mobility$_{i,j}$ and Mobility$_{i,j}\times$ Tel$_{i}$, respectively. Standard errors clustered at the prefecture level are shown in parentheses

$$^{*}p<0.1$$; $$^{**}p<0.05$$; $$^{***}p<0.01$$

**Table 4:** IV estimation with lagged job losses

	Dependent variable: involuntary job separations			
	Persol	Line	DN	DN-JONET
	(1)	(2)	(3)	(4)
Mobility$$_{i,j}$$	− 6.797$$^{*}$$ (3.732)	− 6.096$$^{*}$$ (3.655)	− 9.855$$^{**}$$ (4.373)	− 7.908 (5.565)
Mobility$$_{i,j}\times$$ Tel$$_{i}$$	6.393$$^{*}$$ (3.512)	5.133 (3.115)	27.696$$^{**}$$ (11.821)	14.342 (9.635)
Tel$_{i}$	−0.173 (0.240)	−0.259 (0.202)	− 0.168 (1.150)	− 0.198 (0.886)
Job Loss$$_{i,j-1}$$	0.113 (0.085)	0.103 (0.091)	0.119 (0.079)	0.164$$^{**}$$ (0.080)
Observations	282	282	282	282
R$^{2}$	0.467	0.480	0.502	0.479
F-stat: mobility	31.706	32.656	53.661	115.051
F-stat: mobility $\times$ Tel	189.160	169.398	72.135	314.598

All regressions include prefecture-specific controls and month dummies and are weighted by prefecture population. $\text {Job Loss}_{i,j-1}$ represents the lagged dependent variable. The rows “F-stat: Mobility” and “F-stat: Mobility $\times$ Tel” report the first-stage F-statistics on the relevance of instruments for Mobility$_{i,j}$ and Mobility$_{i,j}\times$ Tel$_{i}$, respectively. Standard errors clustered at the prefecture level are shown in parentheses

$$^{*}p<0.1$$; $$^{**}p<0.05$$; $$^{***}p<0.01$$

Table 1 reports the estimation results of Eq. (16) with the prefecture-month panel data from March to August 2020. We use Persol’s teleworkability index for our baseline regression. The results confirm the negative relationship between mobility and involuntary job loss. In addition, the positive coefficient of the interaction term of mobility and teleworkability indicates that the negative effects of mobility on job losses are mitigated in a prefecture where telework is more prevalent. The results suggest, for example, that Tokyo—the prefecture with the highest teleworkability—suffers less from the state of emergency than other prefectures. Columns (3) and (4) in the table also confirm that stricter non-pharmaceutical policies are positively associated with job losses. The estimates on the interaction term of the policy and teleworkability indexes indicate that teleworkablity weakens this relationship. In Table 2, we try other teleworkablity measures to confirm the mitigation effects of teleworakblity. Overall, the estimated effects are consistent across different measures of teleworkability.

Table 3 shows the estimates of Eq. (16) when instrumenting the mobility index by the policy index, where F-statistics suggest that the policy indexes are highly correlated with the mobility indexes conditional on other controls. The results are qualitatively consistent with those reported in Tables 1 and 2. Notably, the mitigation of the effects through teleworkability is significant and large across all four teleworkability measures. To mitigate a concern for the violation of exclusion restriction of policy choice (e.g., the policy choice may be affected by local economic conditions), Table 4 reports the IV estimates when we include the lagged job loss variable as an additional regressor. The estimates in Table 4 are similar to those in Table 3. Given these results, we conclude that policy-induced mobility changes are associated with job losses and that the teleworkablity of occupations mitigates the negative effects of non-pharmaceutical policies on involuntary job separations. Because our study is based on observational data, however, the result should be interpreted with great caution.

We also examine the robustness for job loss regression by considering an alternative mobility measure of taking the average of retail, grocery, and transit mobility measures excluding workplace mobility measures as $\text {Mobility}_{ij} = (\text {Retail}_{ij}+\text {Grocery}_{ij}+\text {Transit}_{ij})/3.$ As shown in Tables 9, 10 and 11 in the Appendix, we find that the results from using this alternative mobility measure are similar to those from using the mobility measure defined by (19).

## Case growth regression

### Prefecture-daily panel data

The case growth regression (17) is estimated using the prefecture-level daily panel data on confirmed cases. Our sample period is from March 29, 2020, to August 31, 2020. Daily cases for each prefecture are obtained from Dashboard Map of COVID-19 Japan Case (https://gis.jag-japan.com/covid19jp/) while the data on the number of tests is from Toyo Keizai.^9^ Our mobility measures are from COVID-19 Community Mobility Reports by Google,^10^ and we construct the Mobility Index as in (19) but using the daily data. We also collected information on various containment policies (state of emergency, school closure, bar closure, business closure, movie closure, museum closure, nightclub closure) at the prefecture-level from the website of the prefecture governments in Japan.^11^.

We construct the Policy Index as in (20) using the daily data. Our empirical analysis uses 7-day moving averages of daily variables to deal with periodic fluctuations within a week. Table 8 reports the summary statistics of the variables we use for the case growth regression.

**Fig. 6:**
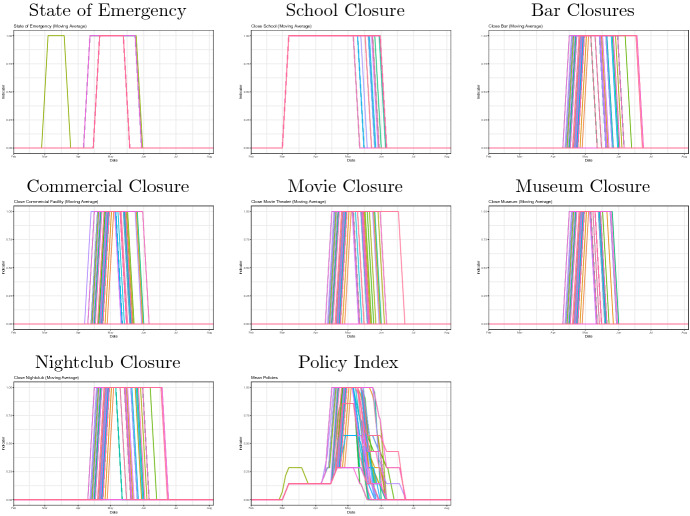
Evolution of containment policies and the policy index at the prefecture-level in Japan

**Fig. 7:**
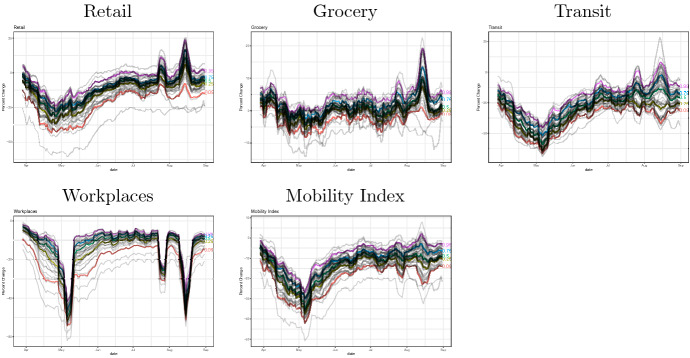
Evolution of mobility and the mobility index at the prefecture-level in Japan

Figure 6 shows the evolution of seven containment policies as well as the policy index at each of 47 prefectures, where each line with different colors represents a prefecture. As shown in Fig. 6, the implementation of containment policies is peaked at the beginning of May, but the timing and the extent of implementation of various containment policies differ across prefectures. Figure 7 shows the evolution of four Google mobility measures (workplaces, retail, grocery, transit) and the Mobility Index at the prefecture level. The mobility sharply decreased in late April, and then the mobility remained low until the mid of May, and then gradually recovered up to the beginning of June. After that, it remained almost stable until the end of August except for several fluctuations due to holidays.

### Regression results

**Fig. 8:**
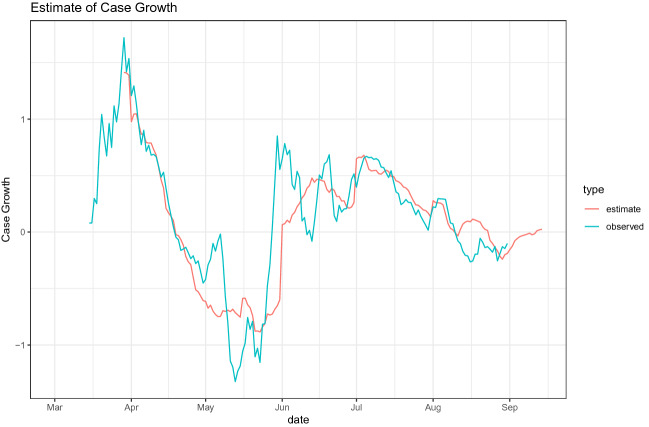
The estimated vs. the observed case growth for Tokyo

Table 5 reports the estimate of (17) with standard errors clustered at the prefecture level. The estimates suggest that an increase in mobility is positively associated with an increase in case growth. The 14 days lagged cases are negatively related to case growth, which likely captures the people’s voluntary behavioral changes (e.g., social distancing, mask-wearing, and handwashing) as a response to a higher transmission risk. An increase in the number of tests is positively related to case growth, although it is not statistically significant. The estimate of 1.233 for the mobility index coefficient implies that the reduction in mobility index in the last week of April in Tokyo relative to the baseline in January and February explains more than one-half of the observed case growth in the second week of May.^12^ Figure 8 shows that the predicted case growth based on the estimates produces a good fit on the actual case growth for Tokyo.

Table 6 shows the estimate of (18) with standard errors clustered at the prefecture level. The estimated coefficient of the Policy index is significantly negative, suggesting that implementation of containment policies reduced people’s mobility. The coefficient of cases is also negatively estimated, indicating that the higher number of cases reduced people’s mobility measured by Google Mobility Reports as people became informed about the prevalence of COVID-19. These results are consistent with the findings from the US using a similar specification (Chernozhukov et al. 2021a, b). Also, people substantially reduced their mobility during the Golden Week period of 2020 in Japan. In combination with the effect of the Golden Week dummy, the estimates imply that the implementation of containment policies has reduced mobility by 19.1 %, explaining 46% of the observed reduction in mobility in Tokyo during the Golden Week.

**Table 5:** The effect of mobility changes on case growth in Japan

Dep. variable	$\varDelta \log \text {Case}_{it}$
Mobility$$_{i,t-14}$$	1.233$$^{***}$$ (0.455)
$$\varDelta \log \mathrm{Case}_{i,t-14}$$	0.188$$^{***}$$ (0.041)
$$\log \mathrm{Case}_{i,t-14}$$	−0.214$$^{***}$$ (0.041)
$\varDelta \log T_{i t}$	0.040 (0.041)
Golden Week$_{t}$	−0.047 (0.265)
Observations	1,704
$R^{2}$	0.421

Weighted by population; prefecture controls, monthly dummies, Golden week dummy, and the log of test included

$$^{*}p<0.1$$, $$^{**}p<0.05$$, $$^{***}p<0.01$$

**Table 6:** The effect of policy changes on mobility in Japan

Dep. variable	Mobility $_{it}$
Policy$$_{it}$$	− 0.079$$^{***}$$ (0.008)
$$\varDelta \log \mathrm{Case}_{i t}$$	0.012$$^{***}$$ (0.002)
$$\log \mathrm{Case}_{i t}$$	− 0.010$$^{***}$$ (0.002)
Golden Week$$_{t}$$	− 0.112$$^{***}$$ (0.005)
Observations	1,704
$R^{2}$	0.932

Weighted by population; prefecture controls, monthly dummies, Golden week dummy included

$$^{*}p<0.1$$, $$^{**}p<0.05$$, $$^{***}p<0.01$$

**Fig. 9:**
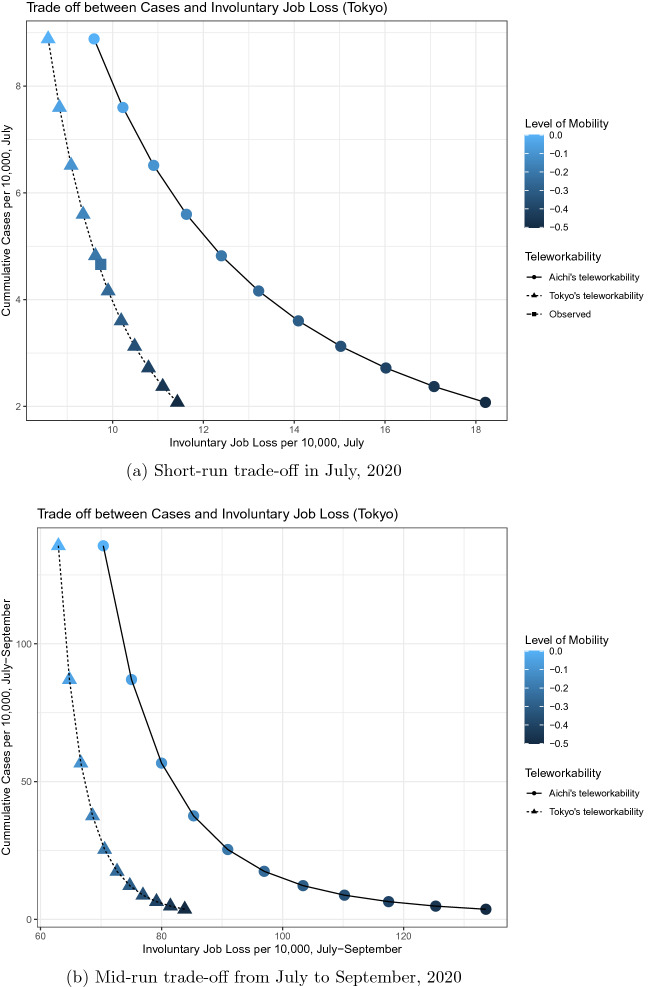
Estimated trade-off between job losses and the number of cases

**Fig. 10:**
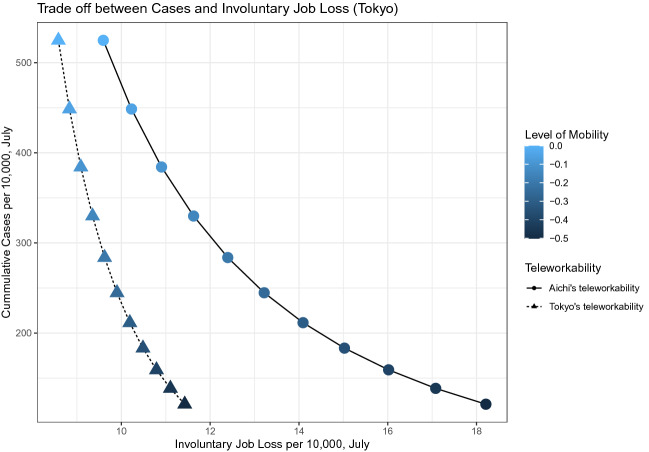
Trade-off between job losses and the number of cases if the number of cases had been high in July, 2020

## Empirical trade-off between job losses and the number of cases

Our empirical findings in Sects. 4 and 5 suggest that people’s movement is the critical determinant for the case growth rate and job losses. If people’s mobility increases, the case growth rate increases, and job losses decreases. By examining how the case growth rate and job losses change as the mobility of people changes, we can quantitatively analyze the trade-off between job losses and the number of cases via mobility changes.

Specifically, taking the estimates of column (1) of Table 3 for job losses and those of Table 5 for case growth, we compute the predicted pair of values for $\varDelta \log \text {Case}_{it}$ and ${ \ln \Big (\frac{Y_{i,j2020}}{Y_{i,j,2019}}\Big )}$ in Tokyo as we change the value of Mobility$_{it}$ and Mobility$_{ij}$ given the Tokyo’s teleworkability value as well as the case number at the end of June and the beginning of July, where the predicted values of $\log \text {Case}_{it}$ and $\varDelta \log \text {Case}_{it}$ are recursively used as the values of covariates to predict the subsequent outcome variables two weeks after.

Then, from these predicted values, we plot a set of possible pairs of values for monthly cumulative cases per 10,000 and involuntary job losses per 10,000 for July in 2020 in Tokyo when the level of job losses were kept at the value of *x*-axis for a month in July under the assumption of ceteris paribus. The result represents an empirical analogue of (14) and is shown in the triangle dots of Fig. 9a.

In Fig. 9a, as the level of mobility decreases from 0 to $-0.5$ (which correspond to a change from the level of February 2020 to the level of mid-May 2020 in Tokyo), the monthly cases per 10,000 decreases from 9 to 2 while the monthly involuntary job losses per 10,000 increase from 8.5 to 11.5. Therefore, the monthly trade-off between cases and involuntary job losses is 2.3 cases for 1 job.

How does the degree of teleworkability affect the trade-off between cases and job losses? Because Tokyo has the highest value of teleworkability index, the impact of decreasing mobility on job losses in Tokyo is smaller than in other prefectures. If the teleworkability of Tokyo had been much lower than the actual value, then Tokyo would have suffered from a higher number of job losses when the mobility decreased in May. To obtain insight into this question, Fig. 9a also presents the trade-off between cases and job losses when the teleworkability of Tokyo had been the same as that of Aichi—about the two-fifth of Tokyo’s value.

As shown in the circle dots of Fig. 9a, if Tokyo had the same teleworkability as Aichi, then, as the level of mobility decreases from 0 to $-0.5$, the monthly cases per 10,000 would decrease from 9 to 2 while the monthly involuntary job losses per 10,000 would increase from 9.5 to 18.5, suggesting that the monthly trade-off between cases and involuntary job losses would have been 0.8 cases for 1 job. This result implies that job losses associated with the decrease in mobility from 0 to $-0.5$ are three times larger in Aichi than in Tokyo because of the difference in teleworkability.

Figure 9b shows the longer-run trade-off between cases per 10,000 and involuntary job losses per 10,000 over the three months from July to September of 2020, where each triangle dot represents the value of monthly cases per 10,000 when the level of job losses were kept at the value of *x*-axis for three months from July to September of 2020. Reflecting the exponential growth of cases when the level of mobility is high, the monthly number of cases per 10,000 from July to Sept of 2020 would have been 275 if the mobility level in Tokyo in July–September of 2020 had been at 0—the same as the mobility level of February of 2020. As the mobility level decreases from 0 to $-0.5$, the number of monthly cases per 10,000 would have decreased from 136 to 4 while the number of monthly job losses per 10,000 would increase from 63 to 83. Here, the implied trade-off between cases and involuntary job losses for the 3-month periods in Tokyo is 6.6 cases to 1 job, which is substantially worse than the short-run trade-off within 1 month.

Figure 10 depicts a set of possible pairs of values for monthly cases and involuntary job losses for July in 2020 in Tokyo as in Figure 9a but now we consider a counterfactual situation in which the number of weekly cases at the beginning of July 2020 were 2520—the highest number of weekly cases recorded in January of 2021 in Tokyo—instead of the actual number of weekly cases, 67. As the triangle dots show, the monthly cases per 10,000 decrease from 525 to 121 while the monthly involuntary job losses per 10,000 increase from 8.5 to 11.5 as the level of mobility decreases from 0 to $-0.5$. Therefore, the monthly trade-off between cases and involuntary job losses would have been 134 cases for 1 jobs, i.e., protecting 1 job requires 134 confirmed cases. As compared to Figs. 9a, 10 shows that the implied trade-off between cases and involuntary job losses deteriorates as the level of the initial value of cases increases.

Our analysis of the trade-off between job losses and the number of cases has important policy implications.

First, understanding the nature of the short-run versus mid-run trade-off is critical to forming effective and rational government policies. For example, when the spread of COVID-19 is limited with a low number of cases, allowing for people to move may not be viewed as costly in terms of health outcomes because the higher mobility does not immediately lead to a substantial number of new infections in the short run. However, if the mobility level is kept uncontrolled or even encouraged, then the number of infected people increases exponentially and may become very high over an extended period. The delayed timing of introducing containment policies causes a high health cost with many infected people. It possibly even induces a risk of many future job losses because of the necessity of implementing stringent containment policies in the future.

Second, due to the nature of the exponential growth of the infection, saving one job becomes quite costly in terms of future infections when the number of current cases is very high. Given concern for the collapse of the healthcare system, the government can not avoid implementing containment policies even if they may lead to more job losses in such a situation. In fact, the Japanese government announced the second state of emergency in January 2021, when Tokyo recorded the highest number of weekly cases.

## Conclusion

Using the Japanese prefecture-level panel data on confirmed cases, involuntary job losses, people’s mobility, and teleworkability for the period from April to August of 2020, this paper quantitatively examines the trade-off relationship between job losses and the spread of COVID-19. Our panel regression analysis indicates that a decline in people’s mobility driven by containment policies such as the declaration of a state of emergency decreased the growth rate of confirmed cases but increased involuntary job losses. We also find that teleworkability mitigates the negative impact of people’s mobility on job losses, suggesting that a prefecture with high teleworkability such as Tokyo would have experienced much higher job losses in May and June of 2020 if Tokyo had low teleworkability.

Using the estimated regressions, we compute the trade-off between job losses and cases by examining how the predicted values of cases and job losses change as we change the values of people’s mobility. The short-run trade-off between cases and involuntary job losses for Tokyo within one month in July of 2020 is estimated as 2.3 cases for 1 jobs per month, i.e., the cost of saving 1 job per month is 2.3 confirmed cases per month. Protecting jobs for a longer period than 1 month is more costly because keeping the high mobility level for an extended period leads to an explosion of the number of confirmed cases due to exponential growth. The longer-run trade-off between cases and involuntary job losses for Tokyo within 3 months from July to September of 2020 is estimated as 6.6 cases for 1 job, i.e., the cost of protecting 1 job per month is 6.6 confirmed cases per month, which is substantially worse than the trade-off within one month. If the number of weekly cases at the beginning of July 2020 were much higher at 2520 instead of the actual number, 67, then the cost of protecting 1 job would have been 134 confirmed cases. Therefore, protecting jobs when the number of cases is very high requires a high health cost.

It is important to emphasize the limitation of our study. First, our study is based on observational data and should be interpreted with caution. In particular, the presence of unobserved confounding factors may invalidate the interpretation of the regression result as causal.

Second, we analyze a limited set of economic and health outcomes. There are a large number of temporary absence from work due to business closure from April to June of 2020 and, therefore, our measure of involuntary job losses only partially reflects the drop in economic activities in the early pandemic period. Furthermore, there are other possible measures of economic activities beyond the measure based on labor inputs, such as consumption-based measures or output-based measures. In terms of health outcomes, the confirmed positive cases in our analysis may not fully capture the infection dynamics, given that the number of tests was said to be limited in the early pandemic period in Japan. Other possible health measures include hospitalizations and deaths. The impact of restricting people’s mobility on mental and physical healths is also an important research topic (e.g., Takaku and Yokoyama 2021).

Third, our analysis does not take into account the differences across different types of individuals. Job loss risk depends on, for example, whether one’s job is regular or non-regular as well as whether their occupations are teleworkable (e.g. Fukai et al. 2021; Hoshi et al. 2021). The elderly and people with pre-existing medical conditions may face a greater health risk from the spread of COVID-19. Therefore, the trade-off between job losses and health outcomes would be different across different individuals. Empirically analyzing the trade-off relationship between economic and health outcomes while considering individual heterogeneity is an important future research topic.

Fourth, our empirical estimate of the trade-off between job losses and the number of confirmed cases captures the trade-off induced by people’s mobility changes under the assumption of ceteris paribus. While we believe that people’s movement is one of the most critical factors to determine both case growth and economic activities in the early pandemic period in Japan, there are other potentially influential factors. For example, a change in people’s precautionary behavior toward the transmission risk (e.g., social distancing, mask-wearing, handwashing) may change the trade-off relationship between job losses and the number of cases, where our empirical analysis only partially addresses this issue by including the past cases as additional controls.

## Figures and Tables

**Fig. 11:**
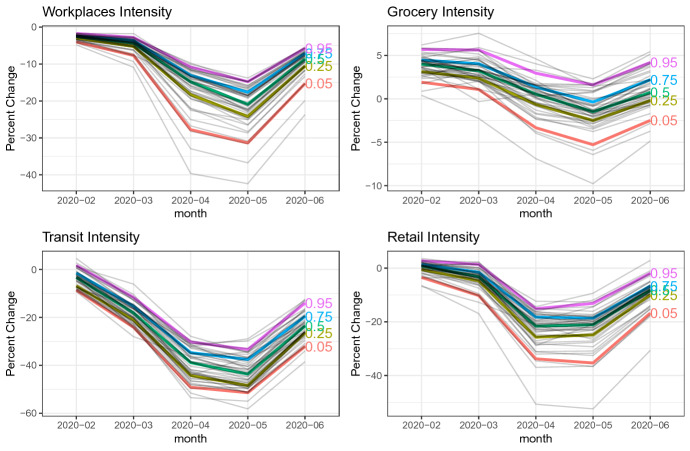
Google mobility reports

## A: Additional figures

See Fig. 11.

## B: Additional tables

See Tables 7, 8, 9, 10, and 11

**Table 7:** Correlation among teleworkability measures, controls and mobility measures

	Dingel-Neiman-uw	Dingel-Neiman-w	Line	Persol	Dingel-Neiman-JONET	Recruit, full-time	Recruit, non-part-time	Recruit, all workers	Population density	Unemployment rate	Poverty rate	Elderly rate	GDP	Secondary sector ratio	Tertiary sector ratio	Social investment	Mob_retail	Mob_Grocery	Mob_Pparks	Mob_Transit	Mob_Workplaces	Mob_Residential	Mob_Mean
Dingel-Neiman-uw	1																						
Dingel-Neiman-w	0.91	1																					
Line	0.77	0.91	1																				
Persol	0.78	0.9	0.96	1																			
Dingel-Neiman-JONET	0.78	0.95	0.9	0.86	1																		
Recruit, full-time	0.55	0.71	0.78	0.75	0.72	1																	
Recruit, non-part-time	0.56	0.72	0.8	0.77	0.73	0.98	1																
Recruit, all workers	0.58	0.7	0.76	0.75	0.69	0.8	0.85	1															
Population density	0.01	− 0.09	− 0.06	− 0.09	− 0.07	0.1	0.03	− 0.04	1														
Unemployment rate	0.33	0.42	0.34	0.28	0.41	0.19	0.24	0.23	− 0.2	1													
Poverty rate	0.06	0.15	0.14	0.12	0.23	0.2	0.24	0.14	0.19	0.5	1												
Elderly rate	− 0.53	− 0.66	− 0.68	− 0.63	− 0.68	− 0.59	− 0.61	− 0.55	0.08	− 0.36	− 0.13	1											
GDP	0.6	0.77	0.8	0.75	0.76	0.64	0.67	0.65	− 0.07	0.29	0.14	− 0.57	1										
Secondary sector ratio	− 0.29	− 0.31	− 0.21	− 0.19	− 0.29	− 0.18	− 0.22	− 0.2	− 0.09	− 0.55	− 0.7	0	− 0.16	1									
Tertiary sector ratio	0.64	0.74	0.62	0.58	0.77	0.48	0.51	0.47	− 0.09	0.65	0.56	− 0.42	0.49	− 0.8	1								
Social investment	0.55	0.62	0.56	0.54	0.55	0.38	0.44	0.47	− 0.21	0.43	0.08	− 0.44	0.79	− 0.28	0.49	1							
Mob_retail	− 0.66	− 0.83	− 0.81	− 0.75	− 0.86	− 0.72	− 0.75	− 0.63	0.04	− 0.38	− 0.33	0.69	− 0.78	0.31	− 0.71	− 0.59	1						
Mob_Grocery	− 0.24	− 0.36	− 0.37	− 0.27	− 0.41	− 0.47	− 0.53	− 0.33	− 0.11	− 0.26	− 0.51	0.51	− 0.39	0.28	− 0.42	− 0.22	0.62	1					
Mob_Pparks	− 0.13	− 0.24	− 0.23	− 0.16	− 0.34	− 0.34	− 0.37	− 0.21	− 0.09	− 0.1	− 0.43	0.43	− 0.18	0.21	− 0.37	0.01	0.56	0.76	1				
Mob_Transit	− 0.37	− 0.5	− 0.48	− 0.36	− 0.56	− 0.47	− 0.49	− 0.27	− 0.02	− 0.39	− 0.26	0.74	− 0.43	0.18	− 0.5	− 0.35	0.67	0.66	0.6	1			
Mob_Workplaces	− 0.75	− 0.91	− 0.96	− 0.91	− 0.9	− 0.75	− 0.76	− 0.71	0.08	− 0.28	− 0.07	0.75	− 0.81	0.1	− 0.57	− 0.57	0.84	0.41	0.27	0.55	1		
Mob_Residential	0.7	0.85	0.92	0.86	0.85	0.78	0.8	0.69	− 0.03	0.17	0.02	− 0.72	0.78	− 0.02	0.49	0.5	− 0.85	− 0.51	− 0.4	− 0.59	− 0.96	1	
Mob_Mean	− 0.64	− 0.81	− 0.81	− 0.72	− 0.85	− 0.71	− 0.73	− 0.57	0.03	− 0.41	− 0.27	0.81	− 0.73	0.23	− 0.67	− 0.55	0.93	0.66	0.57	0.86	0.86	− 0.87	1

**Table 8:** Summary statistics

Statistic	*N*	Mean	St. Dev.	Min	Pctl(25)	Pctl(75)	Max
$\log \text {Case}_{i t}$	1704	3.668	2.104	0.000	2.079	5.407	7.799
$\varDelta \log \text {Case}_{i t}$	1704	0.018	0.839	− 5.697	− 0.444	0.521	3.807
$\varDelta \log T_{i t}$	1704	0.093	0.750	− 6.269	− 0.144	0.272	4.126
$\varDelta \log \ \text {Case}_{i t}$, 14d lag	1704	0.144	0.856	− 3	− 0.4	0.7	4
$\log \text {Case}_{i t}$, 14d lag	1704	3.561	2.035	0.000	2.079	5.136	7.799
log (population)	1704	3.943	0.722	2.101	3.850	4.361	4.929
log (area)	1704	6.263	0.802	4.094	6.267	6.813	6.979
Unemployment rate	1704	1.833	0.553	1	1.8	2	3
Poverty rate	1704	12.333	1.028	11	11.8	13	14
Elderly rate	1704	27.083	2.397	23	25.5	29	31
Golden week	1704	0.035	0.142	0	0	0	1
Policy Index	1704	0.279	0.400	0	0	0.6	1
Mobility Index	1704	− 0.184	0.092	− 0.508	− 0.249	− 0.118	0.080

**Table 9:** Job loss regressions using a mobility index that excludes workplace mobility

	Dependent variable: involuntary job separations			
	(1)	(2)	(3)	(4)
Mobility$$_{i,j}$$	− 1.097$$^{**}$$ (0.542)	− 2.317$$^{**}$$ (0.978)		
Policy$$_{i,j}$$			0.174 (0.120)	0.579$$^{**}$$ (0.292)
Persol$$_{i}$$	− 0.559$$^{**}$$ (0.272)	− 0.256 (0.251)	− 0.504$$^{**}$$ (0.257)	− 0.375 (0.247)
Mobility$$_{i,j}\times$$ Persol$$_{i}$$		2.352$$^{**}$$ (1.018)		
Policy$_{i,j}\times$ Persol$_{i}$				− 0.842$^{*}$ (0.469)
Observations	282	282	282	282
$R^{2}$	0.489	0.497	0.485	0.494

Note. $\text {Mobility}_{ij} = (\text {Retail}_{ij}+\text {Grocery}_{ij}+\text {Transit}_{ij})/3.$ All regressions include prefecture-specific controls and month dummies and are weighted by prefecture population. Standard errors clustered at the prefecture level are shown in parentheses

$$^{*}p<0.1$$; $$^{**}p<0.05$$; $$^{***}p<0.01$$

**Table 10:** Job loss regressions with alternative teleworkability measures

	Dependent variable: involuntary job separations					
	Line	DN	DN-JONET	Line	DN	DN-JONET
	(1)	(2)	(3)	(4)	(5)	(6)
Mobility$$_{i,j}$$	− 2.294$$^{**}$$ (0.978)	− 6.417$$^{***}$$ (2.249)	− 3.854$$^{**}$$ (1.802)			
Policy$$_{i,j}$$				0.507$$^{*}$$ (0.278)	1.606$$^{**}$$ (0.675)	0.898$$^{*}$$ (0.535)
Tel$_{i}$	− 0.296 (0.228)	− 0.901 (1.326)	− 0.038 (1.017)	− 0.381$^{*}$ (0.225)	− 1.679 (1.234)	− 0.284 (0.972)
Mobility$$_{i,j}\times$$ Tel$$_{i}$$	2.013$$^{**}$$ (0.920)	18.203$$^{***}$$ (6.402)	7.636$$^{**}$$ (3.504)			
Policy$$_{i,j}\times$$ Tel$$_{i}$$				− 0.676 (0.417)	− 5.271$$^{**}$$ (2.273)	− 2.190$$^{*}$$ (1.278)
Observations	282	282	282	282	282	282
$R^{2}$	0.501	0.501	0.483	0.496	0.493	0.478

$\text {Mobility}_{ij} = (\text {Retail}_{ij}+\text {Grocery}_{ij}+\text {Transit}_{ij})/3.$ All regressions include prefecture-specific controls and month dummies and are weighted by prefecture population. Standard errors clustered at the prefecture level are shown in parentheses

$$^{*}p<0.1$$; $$^{**}p<0.05$$; $$^{***}p<0.01$$

**Table 11:** Job loss regressions with the policy index as an instrument

	Dependent variable: involuntary job separations			
	Persol	Line	DN	DN-JONET
	(1)	(2)	(3)	(4)
Mobility$$_{i,j}$$	− 7.504$$^{*}$$ (3.974)	− 6.872$$^{*}$$ (3.955)	− 10.975$$^{**}$$ (4.441)	− 8.922 (6.065)
Mobility$$_{i,j}\times$$ Tel$$_{i}$$	6.027$$^{**}$$ (2.998)	4.892$$^{*}$$ (2.640)	29.294$$^{***}$$ (10.781)	15.012$$^{*}$$ (9.046)
Tel$_{i}$	− 0.161 (0.263)	− 0.252 (0.218)	− 0.304 (1.207)	− 0.197 (0.973)
Observations	282	282	282	282
$R^{2}$	0.415	0.432	0.487	0.450
F stat: Mobility	18.369	18.076	28.433	28.957
F stat: Mobility $\times$ Tel	160.170	164.868	42.018	139.511

$\text {Mobility}_{ij} = (\text {Retail}_{ij}+\text {Grocery}_{ij}+\text {Transit}_{ij})/3.$ All regressions include prefecture-specific controls and month dummies and are weighted by prefecture population. Standard errors clustered at the prefecture level are shown in parentheses

$$^{*}p<0.1$$; $$^{**}p<0.05$$; $$^{***}p<0.01$$

## Footnotes

1 Other papers compare the impact of COVID-19 and its heterogeneity across different countries (Adams-Prassl et al. (2020) for the US, the UK, and Germany; Alon et al. (2021) for the US, Canada, Germany, the Netherlands, Spain, and the UK) using microdata from multiple national surveys or real-time surveys.

2 Here, we ignore the possibility that the number of infectious workers affects the aggregate output. Given that the number of infected people in Japan was very small relative to the population in the early pandemic period in Japan, this assumption is reasonable.

3 It is possible to extend the model by introducing multiple types of mobilities such that one type of mobility (e.g., visiting workplaces) is more relevant for the supply side than the other type of mobility (e.g., visiting restaurants), where the latter type may be more relevant for the demand side. In our data, different measures of mobilities are highly correlated each other for our sample period and, therefore, we only focus on one aggregate measure of mobility.

4 Given the data limitation of “teleconsumability,” our empirical analysis focuses on the measure of teleworkability but our empirical result using teleworkability measure likely captures the effect of teleconsumability.

5 The Golden Week is a collection of four national holidays from April 29 to May 5 in Japan.

6 Website: https://www.google.com/covid19/mobility/.

7 Website: https://docs.google.com/spreadsheets/d/1wrWQ0iuKhaNnwUZfi0lBuWEm6mEg3ztNqi6PEvHz7wM/edit?usp=sharing.

8 We map Dingel and Neiman’s measure defined in the O*NET Standard Occupational Classification (O*NET-SOC) to the occupational classification in the Japanese Census in the following steps. First, we map O*NET-SOC to the Standard Occupational Classification (SOC). Then, we map the SOC to the International Standard Classification of Occupation (ISCO). Finally, we map the ISCO to the Japan Standard Occupational Classification (JSOC). Finally, we map the JSOC to the occupational classification in the Japanese Census.

9 https://toyokeizai.net/sp/visual/tko/covid19/.

10 Website: https://www.google.com/covid19/mobility/.

11 This data is made publicly available at https://docs.google.com/spreadsheets/d/1wrWQ0iuKhaNnwUZfi0lBuWEm6mEg3ztNqi6PEvHz7wM/edit?usp=sharing.

12 Because the average reduction in daily mobility during the last week of April in Tokyo is 42.6%, the estimated coefficient of 1.233 implies that the mobility change have decreased the case growth by 0.426 $\times$ 1.233 $=$ 0.523. The actual value of the case growth during the second week of May is about 1.07 so that the mobility change explains $0.523/1.07=56$% of a drop in case growth.
